# Supplementary material for: Physical separation of haplotypes in dikaryons allows benchmarking of phasing accuracy in Nanopore and HiFi assemblies with Hi-C data
Source: Genome Biol. 2022 Mar 25;23:84. doi: 10.1186/s13059-022-02658-2 (PMC8957140; doi:10.1186/s13059-022-02658-2)
Supplement: Supplementary file 1 — Additional file 1: Figures S1-S3. Supplementary figures. Various supplementary figures. [file 13059_2022_2658_MOESM1_ESM.docx]

**
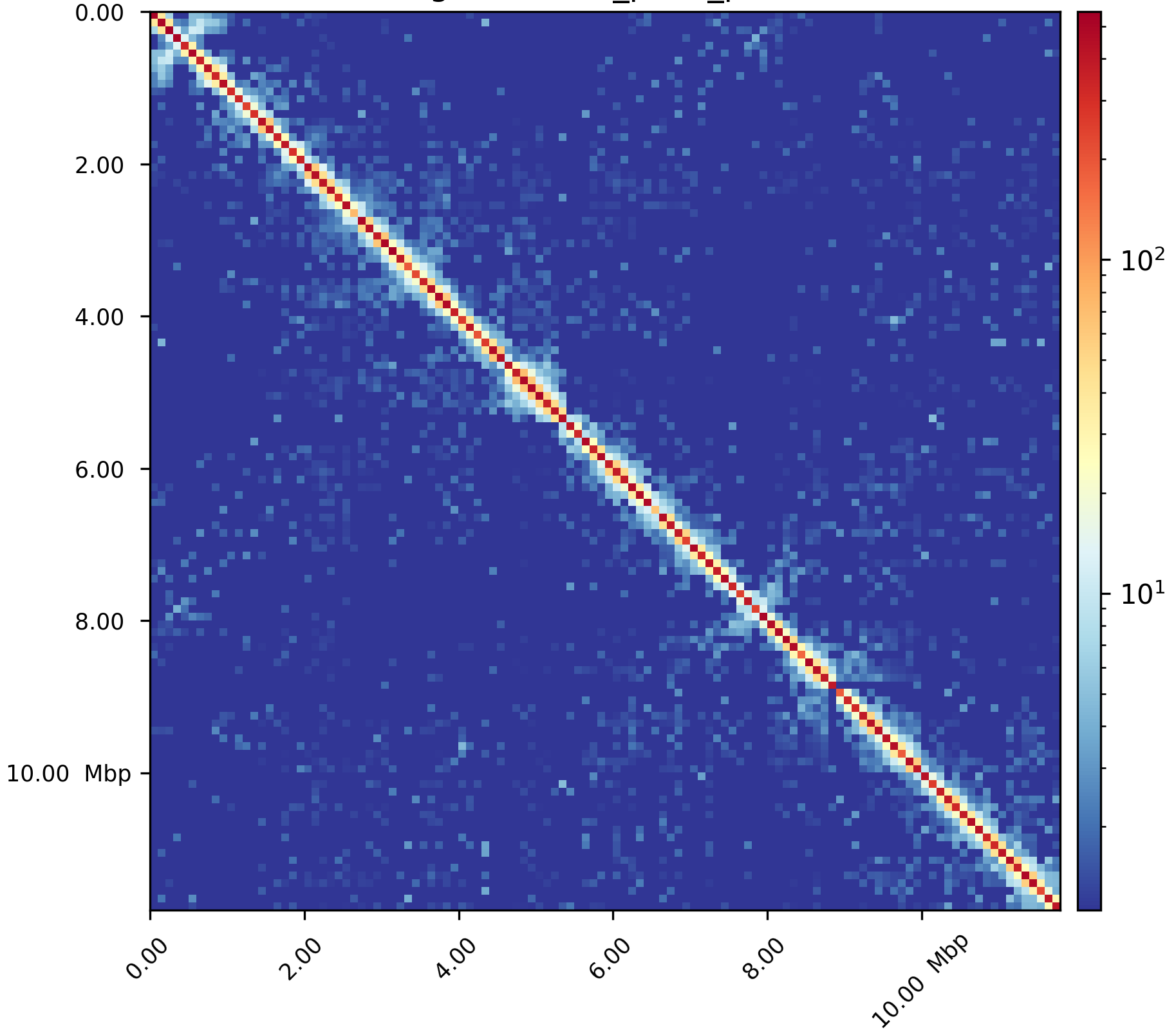
**

**Fig. S1: Hi-C contact map of a chimeric contig in the Nanopore assembly that was broken prior to phasing and scaffolding. (A)** In the Nanopore assembly, contig tig00000001 has two centromeric regions visible at ~0.5 Mb and ~7.8 Mb. The chimeric breakpoint was chosen at 5.35 Mb based on visual inspection of long-read alignments.

**
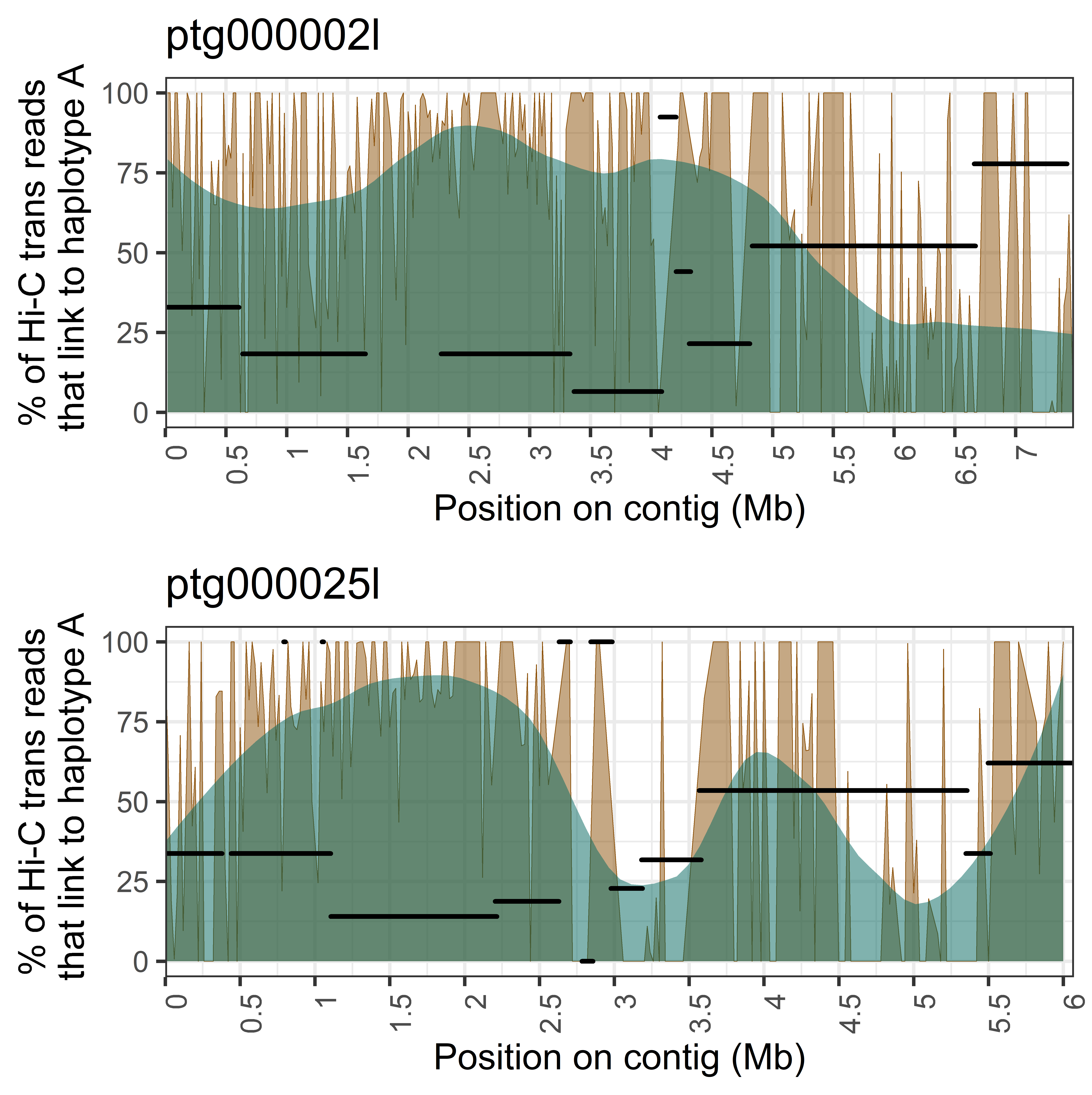
**

**Fig. S2: Contigs ptg000002l and ptg000025l from the HiFi-hifiasm assembly and its associated haplotigs (black segments).** The % of Hi-C *trans*-contacts that link to haplotype A (with an associated smoothing line) are shown. Haplotigs are shown at the *y* coordinate that corresponds to their % of Hi-C *trans-*contacts to haplotype A. If a haplotig has no or only few Hi-C *trans*-contacts, it is shown at *y* = 100. Contig ptg000002l appears to switch phase at ~5.5 Mb, which does not clearly overlap with the corresponding haplotig alignment start and end points. Similarly, the phase switch point at ~4.5 Mb in contig ptg000025l does not clearly overlap with the corresponding haplotig alignment start and end points.


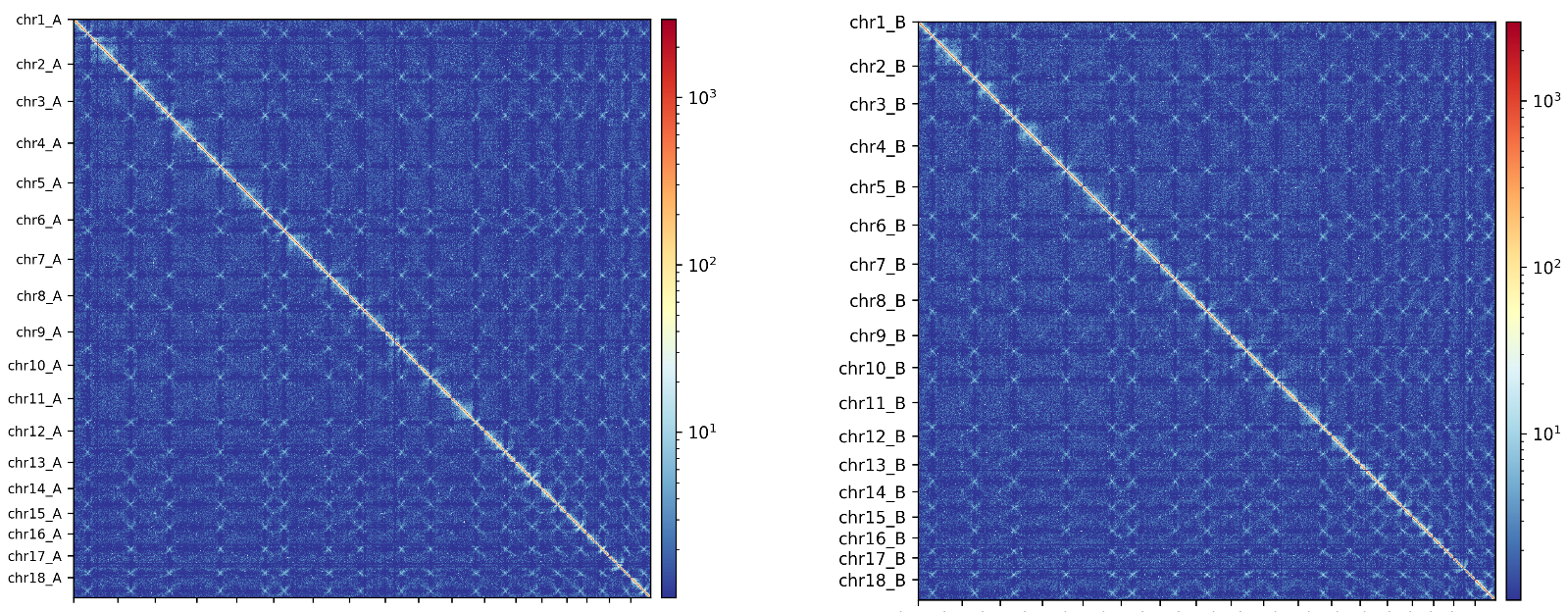


**Fig. S3: Hi-C contact maps of the two chromosome haplotypes in the HiFi assembly.** The centromeres are visible as distinct cross-shapes in the whole haplotype Hi-C contact maps. ​
