# Supplementary material for: Physical separation of haplotypes in dikaryons allows benchmarking of phasing accuracy in Nanopore and HiFi assemblies with Hi-C data
Source: Genome Biol. 2022 Mar 25;23:84. doi: 10.1186/s13059-022-02658-2 (PMC8957140; doi:10.1186/s13059-022-02658-2)
Supplement: Supplementary file 2 — Additional file 2. Review history. [file 13059_2022_2658_MOESM2_ESM.docx]

**1^st^ round**

**Reviewer 1**

This paper by Duan et al is a great study comparing and developing algorithms to assemble long read genomes of non-standard diploid organisms, with the two competing technologies Pacbio and ONT. They took advantage of an organism that has most chromosome haplotypes in separate nuclie. I think that they have done a relatively exhaustive analysis for one study, and produced valuable genome assemblies and knowledge.

My main complaint about the study is that they do not appropriately or sufficiently credit the literature for past findings and source ideas of some of the approaches they used. In other cases, they cite some of the relevant papers, but only as an afterthought in the discussion. Examples are the trio binning approach showing one of the best methods for haplotype phasing (Koren et al 2018), the Vertebrate Genomes Project (VGP) flagship paper showing the importance of haplotype phasing to prevent many of the errors that this study finds (Rhie et al 2021 - initial bioRxiv in 2020), the HiCanu (Nurk et al 2020) and HiFiasm (Cheng et al 2021) papers for the algorithms they use, and the FALCON Phase study being one of the first to incorporate HiC for phasing (Kronenberg et al 2021). Citing these studies as motivators and sources of knowledge of the current study does not diminish the impact of the current study, but show how they move the field forward.

Specific comments:

Background, 1st paragraph. The VGP study by Rhie et al 2021 in Nature should be cited for the source of some of these claims as well.

Results, 1st paragraph. Rhie et al 2020 Mercury paper is cited, but it is not listed in the bibliography. All citations should be doubled checked for their presence in the bibliography.

Same paragraph. Where did they get the kmer analyses idea from? Should be cited.

Results, 2nd paragraph. Cite the source papers for HiCanu and HiFiasm

Is the graph-based approach that the authors developed based on HiFiasm? And is the use of HiC for phasing like FALCON Phase approach? Appears similar.

Figure 6. I am surprised that it is not near 99% separation of haplotypes in the best analyses. FALCON phase with HiC generated 98-99% for vertebrate genomes. In the current study, the average is 80%. Is there a control contig to show when the HiC mapping is clearly for one haplotype versus the other at the > 95% range?

Figure 8. The main text gives the impression that nearly all contigs and scaffolds have been properly separated into their haplotypes when using 40X and 50X Pacbio coverage. But the panels showing the HiCanu assemblies at 40 and 50X have at least 20 scaffolds without complete enough separation. There is one panel with HiFiasm where there is complete separation, but that seems like a control analyses. So, either the text is overstating the claim or is not explaining the results well enough for me to follow the importance of the findings in this figure.

Discussion, 1st paragraph. Some of these citations are the ones that should also be mentioned in the background section and for the motivation of experiments in the results section.

Sentence beginning "HiFi data has been reported to be able" needs a citation. Would be good have page numbers in the resubmission, so reviewers can refer to page numbers.

For this sentence "Whilst this fragmentation of the haplotigs could be an artefact of the assembler used, it might also be due to then error rate of the Sequel technology that is higher than for HiFi sequencing reads." The alternative explanation could be the GA dropout in HiFi reads or false haplotype duplications (see Rhie et al 2021).

Change "We showed that relatively low coverage MinION Nanopore sequencing" to "We showed that relatively equivalent 50X coverage of MinION Nanopore sequencing" since this is equivalent to the amount used for Pacbio sequencing.

Last sentence of discussion. Should cite T2T human genome paper (Nurk et al bioRxiv) and some T2T chromosomes of other vertebrates (Rhie et al 2021).

**Reviewer 2**

The paper "Physical separation of haplotypes in dikaryons allows benchmarking of phasing accuracy in Nanopore and HiFi assemblies with Hi-C data" by Duan et al., takes advantage of the physical separation of haplotypes in the dikaryons with Hi-c technology to assess the correctness of assemblies by ONT and HiFi data. They reached the conclusion that the errors in assembly phasing come from phase switches, which are more abundant in ONT compared to HiFi. Additionally, the authors introduced a new method that uses Hi-C contact graph to phase dikaryotic and correct phase switch errors.

I enjoyed reading the paper, the background section discusses in detail the concept and why they chose rust fungi, with that said I have some concerns:

There is a difference in coverage between ONT and HiFi, while the authors down-sampled HiFi and show the different results, it will be nice if they did it head to head with ONT. Likewise, I would prefer to see some read distribution of raw ONT reads, which plays the main role to identify N50 and assembly it could be associated with figure 1, which has a minor mistake I think (plot C was not mentioned). Moreover, the authors used guppy 4.0 for base-calling, from my experience the last month we published a paper for a framework that calls and phases variants for long-reads, and we saw the same thing, too much switch and consequently, Hamming error comparing ONT to HiFi, but later we found that using guppy 5 changed much; maybe the authors should show that in their paper. Lastly, a minor thing, the authors use long reads and long-reads interchangeably.

**Authors’ response**

Reviewer #1, comment 1: My main complaint about the study is that they do not appropriately or sufficiently credit the literature for past findings and source ideas of some of the approaches they used. In other cases, they cite some of the relevant papers, but only as an afterthought in the discussion. Examples are the trio binning approach showing one of the best methods for haplotype phasing (Koren et al 2018), the Vertebrate Genomes Project (VGP) flagship paper showing the importance of haplotype phasing to prevent many of the errors that this study finds (Rhie et al 2021 - initial bioRxiv in 2020), the HiCanu (Nurk et al 2020) and HiFiasm (Cheng et al 2021) papers for the algorithms they use, and the FALCON Phase study being one of the first to incorporate HiC for phasing (Kronenberg et al 2021). Citing these studies as motivators and sources of knowledge of the current study does not diminish the impact of the current study, but show how they move the field forward.

Response: Apologies for not discussing these studies appropriately in the Introduction. We have re-written parts of the Background section and we have now included the relevant papers as follows:

“The HiCanu and hifiasm assemblers [5,6] take advantage of the high accuracy of HiFi sequencing reads and use a string graph where nodes are reads and edges are overlaps to assemble genomes. Furthermore, these assemblers can use parental data for phasing [5]. Trio binning methods using parental short reads are currently the best methods for generating a pair of haplotype-resolved diploid assemblies [7]. Trio binning first partitions long reads from an offspring into haplotype-specific sets using parental sequencing data for subsequent assembly [7]. However, parental data is not always available and genomic regions that are heterozygous in the parents cannot be phased. Other approaches use k-mers or short reads from Hi-C or strand sequencing data to partition HiFi reads into the haplotypes [8–10]. These methods can struggle to identify haplotype-specific variants in complex or collapsed genomic regions [5]. Falcon-Phase is a recent Hi-C based method for phasing that re-assigns genomic regions that have haplotigs to its phase [11]. It starts with a Falcon-Unzip assembly nomenclature that has a list of contigs with its associated haplotigs and first slices the primary assembly into haplotig regions and collapsed regions. Hi-C data is then mapped and used to assign haplotig pairs to their correct phase with a stochastic algorithm [11]. A limitation of this approach is that it is based on an assembly that might contain collapsed regions and FALCON-Phase will automatically backfill these collapsed homozygous regions in its pseudo-haplotype output. In an evaluation on diploid human assemblies FALCONPhase has a relatively high error rate and swaps large blocks of haplotypes between the two phases [9].”

--------------------------------------------------------

Reviewer #1, comment 2: Background, 1st paragraph. The VGP study by Rhie et al 2021 in Nature should be cited for the source of some of these claims as well.

Response: Apologies for missing to cite this important Vertebrate Genomes Project study by Rhie et al. It is now cited in several places in the Background section.

--------------------------------------------------------

Reviewer #1, comment 3: Results, 1st paragraph. Rhie et al 2020 Mercury paper is cited, but it is not listed in the bibliography. All citations should be doubled checked for their presence in the bibliography.

Response: Apologies for this, we fixed the missing reference and checked/fixed all other references, too.

--------------------------------------------------------

Reviewer #1, comment 4: Same paragraph. Where did they get the kmer analyses idea from? Should be cited.

Response: As suggested we made it clearer that the k-mer analysis is run by merqury:

“The estimated k-mer completeness (fraction of reliable k-mers in the Illumina read set that are also found in the assembly) calculated by merqury [22]” and “an estimated kmer completeness calculated by merqury [22] of 96.4%”.

--------------------------------------------------------

Reviewer #1, comment 5: Results, 2nd paragraph. Cite the source papers for HiCanu and HiFiasm

Response: We added the references as requested.

--------------------------------------------------------

Reviewer #1, comment 6: Is the graph-based approach that the authors developed based on HiFiasm? And is the use of HiC for phasing like FALCON Phase approach? Appears similar.

Response: We now include a discussion of hifiasm and FALCON-Phase in the Background section (see response to comment 1). These two approaches are for denovo genome assembly, whereas our approach is for post-assembly evaluation and phase switch correction in a dikaryon system, without the need for parental data. We acknowledge that the idea of partitioning haplotypes with Hi-C data is broadly common to the Hifiasm, FALCON-Phase and our approach and we now make that clear in the Discussion as follows:

“To overcome this, several strategies have been proposed in diploid genomes such as humans, e.g. trio binning where long reads from an offspring are binned into haplotypespecific sets for subsequent assembly [7] or the partitioning of HiFi reads into the haplotypes with k-mers or short reads from Hi-C or strand sequencing data [8–10]. Here, we build upon this work and introduce a Hi-C contact graph partitioning approach for dikaryons, which is a rapid method that can be run on existing assemblies to phase contigs, to detect phase switches and to evaluate overall phasing accuracy, without the need for parental data.”

--------------------------------------------------------

Reviewer #1, comment 7: Figure 6. I am surprised that it is not near 99% separation of haplotypes in the best analyses. FALCON phase with HiC generated 98-99% for vertebrate genomes. In the current study, the average is 80%. Is there a control contig to show when the HiC mapping is clearly for one haplotype versus the other at the > 95% range?

Response: We now include a Pgt control contig where the Hi-C mapping is in the ~95% range. In these Figure 6 plots, the Hi-C signal is not as clean because phase switches have not been corrected yet. In Figure 7, the clean Hi-C signal is shown and a large proportion of the contigs are in the 95%+ range. To the best of our knowledge the FALCON-Phase evaluation was done with parental trio data, which we do not have for our dikaryons. The Hi-C data will be more noisy than a trio evaluation, for example due to the mapping of paired-end Hi-C reads as single-end reads as well as the difficulty of mapping to repetitive regions such as centromeres. To reflect this, we changed the text as follows:

“We investigated if phase switches cluster in large blocks of genomic regions or if they are randomly distributed along the contigs. For this, we visualized the proportion of Hi- C contacts to haploypes A and B for each scaffold bin. As a control contig, we visualized the Hi-C contacts for the two haplotype sets Bin21_a (contig tig00001246, 1.82 Mb) and Bin21_b (contig tig00001203_02, 1.48 Mb) in the Pgt assembly without phase switches (Figure 6A). Contig tig00001246 has on average 93.6% of its Hi-C contacts to haplotype A, whereas the corresponding haplotig tig00001203_02 has 92.8% of its Hi-C contacts to haplotype B. Thus, the Hi-C signal between scaffold bins is a clear phasing signal. We then investigated the phasing signal and presence of phase switches in the leaf rust assemblies. For example, scaffold bin Bin2 in the HiFi- Canu assembly has two haplotype sets Bin2_a (8 contigs, total 4.43 Mb) and Bin2_b (contig tig00000828, 4.35 Mb).

Figure 6: A contig with its associated haplotigs for each of the three Pt76 assemblies (black segments). The % of Hi-C trans contacts that link to haplotype A (with an associated smoothing line) are shown. Haplotigs are shown at the y coordinate that corresponds to their Hi-C contacts to haplotype A. If a haplotig has no Hi-C contacts, it is shown at at y = 100. (A) Contig tig00001246 from the Pgt assembly without phase switches and its associated haplotig alignment. Contig tig00001246 has on average 93.6% of its Hi-C contacts to haplotype A (indicated with red line), whereas the corresponding haplotig tig00001203_02 has 92.8% of its Hi-C contacts to haplotype B. No phase switches are present. (B) Contig tig00000828 from the HiFi-Canu assembly and its associated haplotig alignments. Contig tig00000828 appears to switch phase at ~1.5-3.7 Mb, which overlaps with the corresponding haplotig alignment start and end points. (C) Contig ptg000011l from the HiFi-hifiasm assembly and its associated haplotig alignments. Contig ptg000011l appears to switch phase at ~3-4.7 Mb and at ~5.8 Mb, which overlaps with the corresponding haplotig alignment start and end points. (D) Contig tig00000001 from the Nanopore assembly and its associated haplotig alignments. Distinct phase switch blocks such as in the HiFi assemblies are not visible. Collapsed regions where the Hi-C contacts are in the 50% range are clearly visible in the Nanopore assembly.”

--------------------------------------------------------

Reviewer #1, comment 8: Figure 8. The main text gives the impression that nearly all contigs and scaffolds have been properly separated into their haplotypes when using 40X and 50X Pacbio coverage. But the panels showing the HiCanu assemblies at 40 and 50X have at least 20 scaffolds without complete enough separation. There is one panel with HiFiasm where there is complete separation, but that seems like a control analyses. So, either the text is overstating the claim or is not explaining the results well enough for me to follow the importance of the findings in this figure.

Response: Apologies if this was unclear. Figure 8 was supposed to illustrate the extent of phase switches in the lower coverage assemblies prior to phase switch correction, compared to the control, fully-phased assembly in the first panel. We added new Figure labels that make it clear that these are assemblies with phase switches still uncorrected. We also added the number of contigs for each plot which contain phase switches to make it clearer that these are not phase switch corrected assemblies yet.

We clarified the text as follows:

“HiFi assemblies below 30x genome coverage have a larger proportion of contigs with phase switches (30x coverage: 43 contigs > 100 Kb; 20x coverage: 106 contigs > 100Kb; Figure 8). Genome coverage of 40x appears to be sufficient to maintain high phasing accuracy within contigs, with only 23 contigs > 100 Kb having phase switches. This is comparable to the original 50x assembly where 17 contigs > 100 Kb have phase switches (Figure 8). This suggests that low coverage HiFi data is not suitable for achieving fully-phased assemblies and that there is also a high coverage limit beyond which phasing of the assembly does not substantially improve.”

Figure 8: Contig coverage of haplotype-specific reads for HiFi assemblies at varying levels of genome coverage. Illumina reads were binned into haplotype-specific reads if they align better to one of the haplotypes from the HiFi chromosomes. These haplotype-specific reads were then re-mapped to the contigs of the downsampled HiFi assemblies as well as to the Nanopore and HiFi-hifiasm assembly. The proportion of each contig covered by reads specific to haplotypes A and B is shown. The HiFi assembly at 40x-50x genome coverage have a clear phasing signal with a relatively low number of contigs containing phase switches, whereas assemblies below 30x coverage have many phase switches.

--------------------------------------------------------

Reviewer #1, comment 9: Discussion, 1st paragraph. Some of these citations are the ones that should also be mentioned in the background section and for the motivation of experiments in the results section.

Response: Yes, thanks for helping us improving the Background section (see comment 1). We now discussed these relevant studies in the Background and discussed implications of our work in the Discussion instead.

--------------------------------------------------------

Reviewer #1, comment 10: Sentence beginning "HiFi data has been reported to be able" needs a citation. Would be good have page numbers in the resubmission, so reviewers can refer to page numbers.

Response: We added a supporting reference for the HiFi haplotype divergence statement as follows: “HiFi data has been reported to be able to separate haplotypes up to a divergence of 0.01% with appropriate genome coverage (https://canu.readthedocs.io/en/latest/faq.html).” We now also include page numbers and line numbers.

--------------------------------------------------------

Reviewer #1, comment 11: For this sentence "Whilst this fragmentation of the haplotigs could be an artefact of the assembler used, it might also be due to then error rate of the Sequel technology that is higher than for HiFi sequencing reads." The alternative explanation could be the GA dropout in HiFi reads or false haplotype duplications (see Rhie et al 2021).

Response: We changed the text as follows:

“In that case, PacBio Sequel data was used to generate a FALCON-Unzip assembly [32]. As reported for vertebrate genomes, FALCON-Unzip can incorrectly retain haplotigs in the primary contig set, which appear as false duplications [2]. The primary FALCON-Unzip assembly has L50 of 2.1 Mb (162 contigs, 92% complete BUSCOs), compared to the haplotig set with L50 of 816 Kb (713 contigs, 84% complete BUSCOs). Whilst this fragmentation of the haplotigs could be an artefact of the assembler used, it might also be due to the error rate of the Sequel technology used in this study [26], which is higher than for HiFi sequencing reads.“

--------------------------------------------------------

Reviewer #1, comment 12: Change "We showed that relatively low coverage MinION Nanopore sequencing" to "We showed that relatively equivalent 50X coverage of MinION Nanopore sequencing" since this is equivalent to the amount used for Pacbio sequencing.

Response: We changed the sentence as requested to:

“We showed that relatively equivalent ~50x haploid genome coverage MinION Nanopore sequencing is not appropriate for achieving haplotype-phased assemblies in the case of leaf rust.”

--------------------------------------------------------

eviewer #1, comment 13: Last sentence of discussion. Should cite T2T human genome paper (Nurk et al bioRxiv) and some T2T chromosomes of other vertebrates (Rhie et al 2021).

Response: Thank you, we added both references to the last sentence of the discussion.

-------------------------------------------------------------------------------------------------------------------

---------------------------

Reviewer #2, comment 1: There is a difference in coverage between ONT and HiFi, while the authors down-sampled HiFi and show the different results, it will be nice if they did it head to head with ONT.

Response: We now include information on the effects of down-sampling of the ONT data as follows:

“Lastly, we also downsampled the Nanopore reads to various levels of haploid genome coverage and assembled them again using Canu. At 40x coverage, the Nanopore assembly has a total size of 222.9 Mb, which is substantially smaller than the estimated haplotype-resolved genome size of ~2*132 Mb (~264 Mb). At 30x genome coverage this declines further to an assembly size of 216.8 Mb (25x genome coverage: 192.7 Mb; 20x genome coverage 185.4 Mb). Thus, these assemblies contain a large amount of collapsed regions and can not be phased.”

--------------------------------------------------------

Reviewer #2, comment 2: Likewise, I would prefer to see some read distribution of raw ONT reads, which plays the main role to identify N50 and assembly it could be associated with figure 1, which has a minor mistake I think (plot C was not mentioned).

Response: We added a read distribution of the ONT and HiFi reads to Figure 1. We fixed the missing mention of plot C in the legend, thanks for noticing.

Figure 1: (A-B) Raw read length histograms for the Nanopore and HiFi reads. The N50 values are indicated with a red line. (C-E) Genome-wide read-depth histograms of the (CA) Nanopore and (DB-E) HiFi assemblies. An assembly with collapsed regions will show a bimodal distribution with a first peak at haploid coverage (indicated by blue line) and a second peak at 2x haploid coverage (indicated by red line). The green line represents the mid-point and genomic regions with coverage above the mid-point are here defined as collapsed regions (Nanopore: 35x coverage; HiFi: 62x coverage). The HiFi assemblies appear to be fully phased whereas the Nanopore assembly has collapsed regions.

--------------------------------------------------------

Reviewer #2, comment 3: Moreover, the authors used guppy 4.0 for base-calling, from my experience the last month we published a paper for a framework that calls and phases variants for long-reads, and we saw the same thing, too much switch and consequently, Hamming error comparing ONT to HiFi, but later we found that using guppy 5 changed much; maybe the authors should show that in their paper.

Response: We re-basecalled the Nanopore data with Guppy 5 as suggested by the reviewer. We included a new Section which describes these results as follows:

“Re-basecalling the Nanopore reads with Guppy version 5 slightly improves phasing accuracy

Recently an updated version of the ONT Guppy basecaller became available (Guppy v5) and we used it to re-basecall the MinION fast5 reads. This resulted in a total of 6.0 Gb of Nanopore Guppy v5 reads (L50 of reads: 31.0 Kb), which were assembled using Canu [28], and subsequently polished. This process yielded an assembly that is very similar to the Guppy v4 assembly (Table 1), comprising 798 contigs with total size of 234.8 Mb and N/L50 of 44/927.4 Kb. A long-read coverage analysis estimated that in the Nanopore Guppy v5 assembly ~35.5 Mb are collapsed genomic regions (data not shown). We used Illumina read mapping and SNP calling against the Nanopore Guppy v4 and Nanopore Guppy v5 genome assemblies to assess the accuracy of each assembly. The Nanopore Guppy v4 assembly had ~300 SNPs per Mb whereas the Guppy v5 assembly has higher accuracy of ~176 SNPs per Mb (HiFi-Canu: ~7 SNPs per Mb). As expected, heterozygous SNPs are enriched in collapsed regions (Nanopore Guppy v4: 695.2 SNPs per Mb; Nanopore Guppy v5: 636.0 SNPs per Mb; HiFi-Canu: 6.9 SNPs per Mb). Homozygous SNPs in non-collapsed regions indicate assembly accuracy, and in these regions the Nanopore Guppy v4 assembly has ~132 SNPs per Mb whereas the Nanopore Guppy v5 assembly has ~38 SNPs per Mb (~4 SNPs per Mb for the HiFi-Canu assembly).

We then investigated the accuracy of generating haplotypes assemblies using each basecaller (Nanopore Guppy v4 vs. Nanopore Guppy v5). For this, we applied the phasing pipeline to the Nanopore assemblies. For the Nanopore Guppy v4 assembly, the two haplotypes comprised 118.6 Mb and 83.8 Mb, respectively. For the Nanopore Guppy v5 assembly, the two haplotypes comprised 109.3 Mb and 88.9 Mb, respectively. Whilst we found before that the HiFi-Canu and HiFi-hifiasm exhibit similar phasing profiles to the Pgt raw assembly with only few phase switches (Figures 4 and 5), the Nanopore assemblies did not show a clear phasing signal, with most contigs of haplotype A showing Hi-C links to haplotype B and vice versa (Figure 9). In the Nanopore Guppy v4 assembly we found that a high proportion (58.8%) of transcontacts occur between haplotypes. This indicates that extensive phase swapping occurs in the Nanopore assembly. The Nanopore Guppy v5 assembly has less phase switches present, with a lower proportion of 47% of trans-contacts occuring between haplotypes. However, this is still a substantially higher proportion of false positive trans-contacts than in the HiFi-Canu assembly, where only 8.6% of trans-contacts are between haplotypes. Taken together, whilst re-basecalling of the Nanopore data with Guppy v5 improves the accuracy of the assembly, there are still a high proportion of phase switches present which precludes phasing of the assembly.

--------------------------------------------------------

Reviewer #2, comment 5: Lastly, a minor thing, the authors use long reads and longreads interchangeably.

Response: We fixed this and changed long read sequencing to long-read sequencing throughout the manuscript.

**2^nd^ round**

**Reviewer 2**

I would like to thank the authors for their work. All my questions and concerns have been answered.
